# The Influence of Zeolite (Sokyrnytsya Deposit) on the Physical and Chemical Resistance of a Magnesium Potassium Phosphate Compound for the Immobilization of High-Level Waste

**DOI:** 10.3390/molecules24193421

**Published:** 2019-09-20

**Authors:** Svetlana A. Kulikova, Sergey E. Vinokurov

**Affiliations:** Vernadsky Institute of Geochemistry and Analytical Chemistry, Russian Academy of Sciences, 19 Kosygin st., Moscow 119991, Russia; vinokurov.geokhi@gmail.com

**Keywords:** zeolite, magnesium potassium phosphate compound, cesium, strontium, plutonium, americium, immobilization, compressive strength, leaching

## Abstract

The manuscript presents the results of the development of new material for high-level waste (HLW) management: the magnesium potassium phosphate (MKP) compound. The possibility of using zeolite (Sokyrnytsya deposit) to increase the mechanical, thermal, and hydrolytic resistance of this compound with immobilized HLW was studied. The main component of the used natural zeolite is a mineral of the clinoptilolite–heulandite series, and quartz, microcline, and clay minerals (illite, sepiolite, and smectite) are present as impurities. The compressive strength of the compound, containing at least 4.2 wt % zeolite, is about 25 MPa. Compound containing 28.6 wt % zeolite retains high compressive strength (at least 9.0 MPa), even after heat treatment at 450 °C. The adding of zeolite to the composition of the compound increases its hydrolytic stability, while the leaching rate of the mobile nuclides ^137^Cs and ^90^Sr decreases up to one order of values. Differential leaching rate of radionuclides from the compound containing 28.6 wt % zeolite is 2.6 × 10^−7^ for ^137^Cs, 2.9 × 10^−6^ for ^90^Sr, 1.7 × 10^−9^ for ^239^Pu, and 2.9 × 10^−9^ g/(cm^2^∙day) for ^241^Am. Thus, the properties of the resulting compound correspond to the requirements for solidified HLW in Russia.

## 1. Introduction

The nuclear fuel cycle is the main source of radioactive waste (RW) and produces all types of it, including high-level waste (HLW). HLW is formed as a result of reprocessing of spent nuclear fuel (SNF) from reactor plants, and consists of fuel components residue (U, Pu), minor actinides (Am and Cm isotopes), fission products (Cs, Sr, etc.), corrosion products (Fe, Cr, Al, Mo, Ni, Zr, etc.) and process contaminants (Na, K, Ca, Mg, etc.). According to the concept developed by the International Atomic Energy Agency, RW of any activity level must be immobilized—that is, transformed into a waste form by solidification, embedding, or encapsulating [1].

Vitrification is the only industrial technology for HLW management [2]. The use of the magnesium potassium phosphate (MKP) compound based on the MgKPO_4_ × 6H_2_O matrix has particular interest as an alternative to aluminophosphate or borosilicate glass. Unlike glass, the MKP compound is a mineral-like material (K-struvite) [3], forming at room temperature and atmospheric pressure [4]. The influence of different HLW types on the properties of the MKP compound was studied, including ^99^Tc waste solutions [5], Pu contaminated ash [6], surrogate denitrated HLW from the PUREX process [7], and HLW bottom sediment surrogates [8]. In our early works, we confirmed the premise of using the MKP compound for HLW solidification of various composition and origin, including historical waste from the implementation of military programs of the Soviet Union and the United States [9,10], as well as waste obtained after the reprocessing of SNF of a 1000 MW water–water energetic reactor (WWER-1000) [11,12,13].

The main quality indicators of matrix materials for HLW conditioning are mechanical, thermal, and hydrolytic stability [14]. Thermal stability is required for possible significant heating (up to 300–400 °C) of the compound, due to heat release of radionuclides of HLW, and is assessed by the mechanical strength of the compounds after heat treatment. Hydrolytic stability is estimated by the leaching rate of radionuclides (^137^Cs, ^90^Sr, ^239^Pu) from the compounds. The activity of HLW is mainly determined by the isotopes of cesium (^134^Cs, ^137^Cs), which is an alkali metal and, therefore, the most leached from the matrix materials. It is known that to reduce the leaching of Cs isotopes from matrices, radionuclides are converted to insoluble compounds (for example, as part of nickel–cesium ferrocyanide [12]) or exposed by sorption on specially selected sorbents (based on clinoptilolite [15,16,17,18] and clay minerals [19,20]). Inorganic sorbents based on transition metal ferrocyanides are unstable in alkaline media at pH 10 [1] and elevated temperatures.

It was previously noted that increasing the temperature of the MKP compound may lead to a decrease of its stability [12,13]. Zeolite is a promising mineral modifier of the MKP compound. Zeolites are porous, hydrated aluminosilicates with a general formula of M_x/m_[(AlO_2_)_x_(SiO_2y_)], where M_x/m_ designates an ion-exchangeable cation [21]. Zeolites are the preferred inorganic ion exchange materials for radionuclide (especially cesium) concentrations from liquid RW, because of their radiation stability, high selectivity, and cation exchange capacity [22,23]. They can be incorporated into matrices (for example, cement [19,24] and glass [25,26]) ensuring a higher degree of radionuclide retention [1]. However, in the literature, there are no data on the effectiveness of the zeolite in the MKP compound in the HLW immobilization.

In this work, we study the influence of zeolite on the physical and chemical properties of the MKP compound, in order to provide the mechanical, thermal, and hydrolytic resistance required for solidified HLW.

## 2. Results and Discussion

### 2.1. Mineral and Chemical Composition of Zeolite

**Figure 1:**
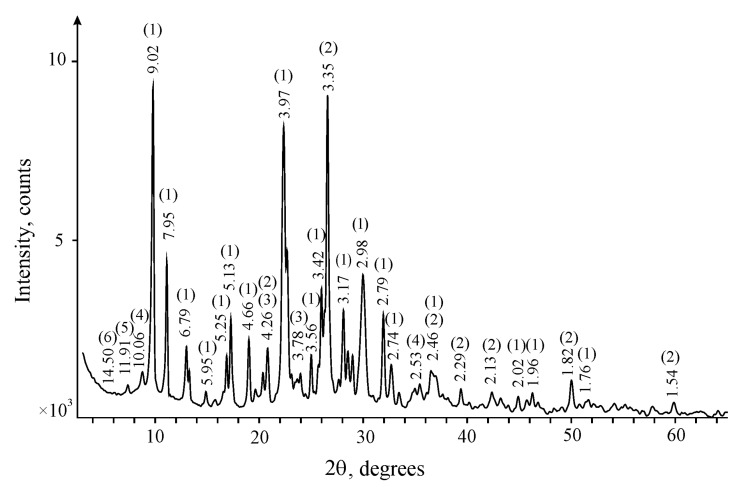
X-ray diffraction pattern of the zeolite sample. (1) zeolites, (2) quartz, (3) microcline, (4) illite, (5) sepiolite, and (6) smectite.

The obtained data on the study of the mineral and chemical composition of the zeolite samples are given in Figure 1 and Table 1, respectively. Zeolites (clinoptillolite and heulandite) were identified by a series of basic reflexes (9.02 Å, 7.95 Å, 6.79 Å, 5.95 Å, 5.25 Å, 5.13 Å, 4.66 Å, 3.97 Å, etc.), quartz (4.26 Å, 3.35 Å, 2.46 Å, 2.29 Å, 2.13 Å, 1.82 Å, 1.54 Å), microcline (4.26 Å, 3.78 Å, etc.), illite (10.06 Å, 2.53 Å, etc.), sepiolite (11.91 Å) and smectite (14.50 Å) (Figure 1). It was established that the main component of the used zeolite is a mineral of the clinoptilolite–heulandite series (74%), which is consistent with the data that deposits of zeolite tuff in this region are characterized by clinoptilolite content from 60% to 75% [27,28]. Quartz (12.0%), microcline (2.0%), and clay minerals—illite (5.0%), sepiolite (5.0%), and smectite (2.0%)—are present in the rock as impurities. According to the results of X-ray fluorescence analysis, it was determined that the sample consists of silicon and aluminum oxides; the content of SiO_2_ and Al_2_O_3_ is 78.165 and 11.530 wt % (Table 1), respectively. Oxides of Na, K, Mg, Ca, Fe, and Ti are also present, and SrO, MnO, P_2_O_5_, Rb_2_O, BaO, ZrO_2_, and Y_2_O_3_ were found as micro-impurities.

### 2.2. Mechanical and Thermal Stability of the Magnesium Potassium Phosphate Compound

Data on the optimal zeolite filling of the MKP compound samples were given in Table 2. It was found that the compressive strength of the compound increases with the addition of zeolite in the composition of the samples (Table 2), while the strength does not increase with the injection of zeolite more than 4.2 wt %. Clearly, this amount of zeolite fills the pores in the MKP compound, which make up about 5 vol % [4].

It was noted that after holding the compounds at 450 °C for 4 h, the samples lost their compressive strength, with the exception of samples containing at least 28.6 wt % zeolite (compressive strength was 9.7 ± 0.5 MPa). The obtained values for the compressive strength of the samples meet the requirements for vitrified HLW (at least 9.0 MPa) [14]. It is likely that zeolite under significant content in the compound exhibits reinforcing properties, which allows the maintenance of the strength of the compound at the required level. It should be noted that the effect of increasing the strength of the cement compound with an optimal content of about 30% zeolite was previously shown [29]. Thus, the MKP compound containing 28.6 wt % zeolite was selected to study hydrolytic stability.

### 2.3. Hydrolytic Stability of the Magnesium Potassium Phosphate Compound

The specific activity of the radionuclides in the compound is presented in Table 3. The content of the HLW surrogate in the compound samples was about 31.6 wt %.

Samples of the MKP compound obtained by solidification of the HLW surrogate with the addition of zeolite (MKPZ), and after heat treatment of the compound at 120 °C (MKPZ_term), were used to study hydrolytic stability.

**Figure 2:**
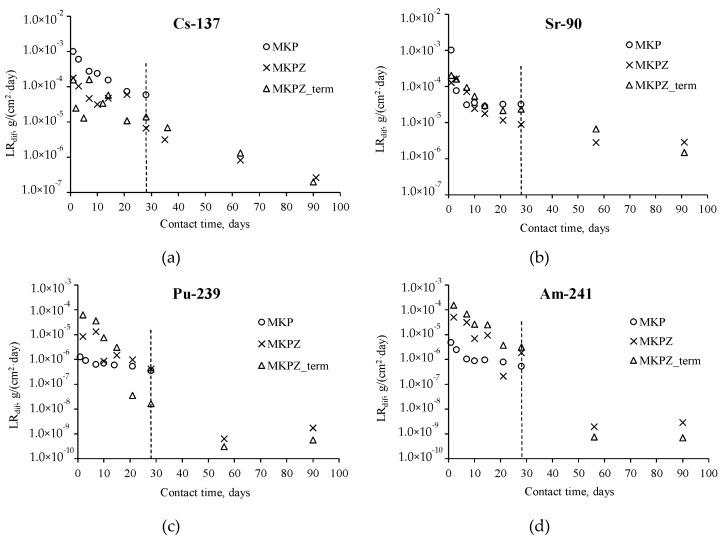
Kinetic curves of the radionuclides leaching from the MKP compound not containing zeolite (MKP) and containing zeolite (MKPZ), including after heat treatment of the MKPZ compound (MKPZ_term): ^137^Cs (**a**), ^90^Sr (**b**), ^239^Pu (**c**), ^241^Am (**d**).

The leaching degree (*E*), differential (*LR_dif_*) and integral (*LR_int_*) leaching rate of radionuclides from the synthesized compound were determined in accordance with standards [30]. The kinetic curves of the radionuclide *LR_dif_* from the MKP compounds are shown in Figure 2a–d, and the E and *LR_int_* are presented in Table 4. To compare the hydrolytic stability of the compounds, the curves of the radionuclides *LR_dif_* from the MKP compounds not containing zeolite are also shown in Figure 2a–d.

Previously, it was found that the differential leaching rate of ^239^Pu and ^241^Am from the MKP compound not containing zeolite on the 28th day of contact with water is 3.5 × 10^−7^ and 5.3 × 10^−7^ g/(cm^2^∙day), respectively (Figure 2c,d) [11], while for ^137^Cs and ^90^Sr it is 5.8 × 10^−5^ and 3.2 × 10^−5^ g/(cm^2^∙day), respectively (Figure 2a,b), in the present work.

It was shown that adding zeolite to the composition of the compound increases hydrolytic stability, while the leaching rate of ^137^Cs decreases by one order of values (Figure 2a), and that of ^90^Sr by four times (Figure 2b). The effect of zeolite on the leaching of ^239^Pu and ^241^Am is insignificant (Figure 2c,d), and the leaching of these radionuclides is at a low level. The differential leaching rate of radionuclides from the compounds containing 28.6 wt % zeolite on the 90th day of contact with water was 2.6 × 10^−7^ for ^137^Cs, 2.9 × 10^−6^ for ^90^Sr, 1.7 × 10^−9^ for ^239^Pu, and 2.9 × 10^−9^ g/(cm^2^∙day) for ^241^Am. It should be noted that the leaching rate of radionuclides on the 90th day does not change, even after heat treatment of the compounds at 120 °C (Figure 2, Table 4). Thus, the hydrolytic stability of the MKP compound corresponds to the requirements of glass-like compound application for HLW immobilization [14] in Russia (according to the requirements, the leaching rate of ^137^Cs, ^90^Sr, and ^239^Pu is ≤ 1.0 × 10^−5^, 1.0 × 10^−6^, and 1.0 × 10^−7^ g/(cm^2^∙day), respectively).

## 3. Materials and Methods

### 3.1. Chemicals and Procedures

The experiments were performed in the glove box (Pererabotka, Dzerzhinsk, Nizhny Novgorod region, Russia) at ambient atmospheric conditions. The chemicals used in the experiments were of no less than chemically pure grade.

The samples of the MKP compound were prepared at room temperature by solidification of the industrial HLW surrogate, obtained after the SNF reprocessing of the WWER-1000, according to the procedure previously given [12]. The HLW surrogate was prepared by dissolving the metal nitrates in an aqueous solution of nitric acid; nuclides were added to the HLW surrogate individually. The content of HNO_3_ in the HLW surrogate was 3.2 mol/L, and the density was about 1.21 kg/L. Preliminary preparation of the HLW surrogate and binding components (MgO, KH_2_PO_4_) was previously reported [11,31]. The chemical and radionuclide composition of the prepared HLW surrogate is presented in Table 5. The density of the HLW surrogate is 1280 g/L, the pH was 7.0 ± 0.1, and the salt content was about 484 g/L.

The natural zeolite of the Sokyrnytsya deposit of the Transcarpathian region (ZEO-MAX LLC, Russia), with a particle size of 0.07–0.16 mm and a specific surface area of 17.5 m^2^/g, was used as a mineral modifier.

As a result, cubic samples of the MKP compound with dimensions of 2 cm × 2 cm × 2 cm were prepared. The samples were kept for at least 15 days at ambient atmospheric conditions.

### 3.2. Methods

The mineral composition of zeolite was calculated using the powder X-ray diffraction (XRD) method (Ultima-IV, Rigaku, Tokyo, Japan). The X-ray diffraction data were interpreted using the specialized Jade 6.5 program package (MDI, Livermore, CA, USA), with the PDF-2 powder database. The mineral composition was determined using the Rietveld method [32], with a PROFEX GUI software package for BGMN [33]. The elemental composition of the zeolite was studied using an X-ray fluorescence spectrometer (Axious Advanced PW 4400/04, PANalytical B.V., Netherlands).

The compressive strength of the obtained compounds was determined using a universal test machine AG-X Plus (Shimadzu, Japan), in accordance with GOST 310.4-81 [34].

Thermal stability of the obtained compounds was studied after heat treatment to constant weight, both at 120 °C during 24 h (heating rate 4 °C/min), for quantitative removal of the bound water from the compounds (as previously shown in reference [35]), and at 450 °C during 4 h (heating rate 7 °C/min), in accordance with the current requirements for solidified HLW [14]. Compound samples were heat-treated in the muffle furnace (SNOL 30/1300, AB UMEGA GROUP, Utena, Lithuania); cooling of the samples occurred in a non-functioning furnace with its direct cooling.

The initial compounds and the samples after removal of the bound water were tested for hydrolytic stability. The hydrolytic stability of compounds was determined using the semidynamic test, in accordance with GOST R 52126-2003 [30]. The conditions were as follows: monolithic compound, a leaching agent of bi-distilled water (pH 6.6 ± 0.1, volume 200 mL), and temperature at 25 ± 3 °C. Periodic replacement of the leaching agent was proceeded after 1, 3, 7, 10, 14, etc. days, with the total duration of the test up to 90 days (28 days for compounds not containing zeolite). The content of radionuclides in solutions after leaching was determined by radiometric methods (α- and γ-ray spectrometers; Canberra, Meriden, CT, USA, and a α-β radiometer UMF 2000, LLC RPE «Doza», Zelenograd, Moscow, Russia). The calculation procedure of the differential (*LR_dif_*) and integral (*LR_int_*) leaching rates of radionuclides from samples is given in [31].

## 4. Conclusions

As a result of the study, it is shown that the use of zeolite from Sokyrnytsya deposit as a mineral modifier can increase the mechanical strength (two times), as well as the thermal (up to 450 °C) and hydrolytic resistance to leaching of HLW radionuclides (the leaching rate of ^137^Cs decreases by one order of values, and ^90^Sr by four times). These conclusions are explained by the properties of zeolites, which have reinforcing properties and high adsorption ability to radionuclides (especially cesium). Thus, the MKP compound containing such zeolite can be recommended to radiochemical plants as a new material for HLW immobilization.

## Figures and Tables

**Table 1:** Chemical composition of zeolite sample, according to data of X-ray fluorescence analysis.

Compound Formula	Concentration (%)	Compound Formula	Concentration (%)
SiO_2_	78.165	SrO	0.053
Al_2_O_3_	11.530	MnO	0.050
Na_2_O	3.012	P_2_O_5_	0.039
K_2_O	2.232	Rb_2_O	0.022
MgO	2.054	BaO	0.015
CaO	1.622	ZrO_2_	0.009
Fe_2_O_3_	0.984	Y_2_O_3_	0.001
TiO_2_	0.212	Total	100.000

**Table 2:** Effect of zeolite on the compressive strength of the magnesium potassium phosphate (MKP) compound.

Zeolite Content (wt %)	Compressive Strength (MPa)
0	12.0 ± 3.0
4.2	23.8 ± 1.7
16.7	26.6 ± 4.0
23.0	22.3 ± 2.0
28.6	25.6 ± 3.4

**Table 3:** Characteristic of the MKP with zeolite (MKPZ) compound prepared under study.

Radionuclide	Specific Activity (Bq/g)
^137^Cs	1.7 × 10^4^
^90^Sr	1.5 × 10^4^
^239^Pu	6.8 × 10^4^
^241^Am	9.3 × 10^3^

**Table 4:** Integral leaching rate (*LR_int_*) and leaching degree (*E*) of radionuclides from the MKPZ compound, according to standard [30], on the 90th day of contact with water.

Radionuclide	Compound	*LR_int_* (g/(cm^2^∙day))	*E* (%)
^137^Cs	MKPZ	1.5 × 10^−5^	0.22
	MKPZ_term	1.2 × 10^−5^	0.22
^90^Sr	MKPZ	1.3 × 10^−5^	0.19
	MKPZ_term	1.9 × 10^−5^	0.34
^239^Pu	MKPZ	1.1 × 10^−6^	0.02
	MKPZ_term	3.8 × 10^−6^	0.07
^241^Am	MKPZ	3.8 × 10^−6^	0.05
	MKPZ_term	1.0 × 10^−5^	0.18

**Table 5:** Radionuclide and chemical composition of the prepared high-level waste (HLW) surrogate.

Specific Activity of Actinides (Bq/L)	Metal Content (g/L)
^239^Pu: 2.8 × 10^8^ ^241^Am: 3.8 × 10^7^ ^137^Cs: 7.1 × 10^7^ ^90^Sr: 5.8 × 10^7^	Na – 83.9; Sr – 3.0; Zr – 5.6; Mo – 0.8; Pd – 4.1; Cs – 7.4; Ba – 1.2; Nd – 28.2; Fe – 0.8; Cr – 2.3; Ni – 0.4; U – 2.1
